# Gender-sensitive sentiment analysis for estimating the emotional climate in online teacher education

**DOI:** 10.1007/s10984-022-09405-1

**Published:** 2022-02-01

**Authors:** Mireia Usart, Carme Grimalt-Álvaro, Adolf Maria Iglesias-Estradé

**Affiliations:** 1 Department of Pedagogy, Universitat Rovira I Virgili, Tarragona, Spain; 2 Computer, Multimedia and Telecommunication Studies, Universitat Oberta de Catalunya, Barcelona, Spain

**Keywords:** Gender, Online learning, Sentiment analysis, Teacher training, Virtual learning environments

## Abstract

Teacher training takes place in distance education to a large extent. Within these contexts, trainers should make use of all the information available to adapt and refine their instructional methods during the training process. Sentiment analysis (SA) can give immediate feedback of the emotions expressed and help in the training process, although it has been used infrequently in educational settings, slow to assess, and bound to interpretative issues, such as gender bias. This research aimed to design and evaluate a SA gender-sensitive method as a proxy to characterize the emotional climate of teacher trainees in an online course. An explanatory case study with mixed methods was implemented among students of the Interuniversity Master of Educational Technologies (*N* = 48). Participants’ messages were analyzed and correlated with learning achievement and, along with a qualitative study of participants’ satisfaction with the Master’s degree, to validate the effectiveness of the method. Results show that sentiment expression cannot be used to exactly predict participants’ achievement, but it can guide trainers to foresee how participants will broadly act in a learning task and, in consequence, use SA results for tuning and improving the quality of the guidance during the course. Gender differences found in our study support gendered patterns related to the emotional climate, with female participants posting more negative messages than their counterparts. Last but not least, the design of well-adjusted teaching–learning sequences with appropriate scaffolding can contribute to building a positive climate in the online learning environment.

## Introduction

Preservice and inservice teacher training courses in some countries are mainly implemented in online learning modalities (Philipsen et al., 2019). In these online learning environments, according to Buckingham Shum and Ferguson (2012), trainees interact to clarify their intentions, ground their learning, engage in meaningful conversations, and receive feedback from their trainers. In the COVID-19 context, the use of virtual learning environments (VLE) has become even more widespread, leading educators to re-conceptualize fundamental issues of teaching (Crawford et al., 2020). These changes probably will have lasting effects on learning environments in higher education and will need to be reviewed and adapted to the new normality (Khan & Jawaid, 2020).

The emotional climate of a group, defined as the quality of the social and emotional interactions between trainees, their peers, and trainers (Reyes et al., 2012), is a key element for creating safe and creative learning environments (Alonso-Tapia & Nieto, 2019), because it can influence trainees’ motivation to engage in learning within a particular classroom environment (Fraser et al., 2021). Consequently, how trainers manage the group’s emotional states can affect not only trainees’ emotional outcomes, but also their learning and success in the course (Alonso-Tapia & Nieto, 2019). From the perspective of formative assessment, it is crucial to have an accessible measurement of the emotional climate of a group so that trainers can adapt and refine their instructional methods as the course goes on. Although several instruments for characterizing a group’s emotional climate can be found in the literature, these instruments are based on observational tools and questionnaires (Fraser et al., 2021). To improve the quality of formative assessment, it might become necessary to explore other methods for characterizing the emotional climate of a group which, in online learning environments, could involve studying trainees’ exchanges of textual data. This information could therefore be used by trainers to improve teaching and learning processes in general (Pinger et al., 2018).

Nevertheless, analyzing textual data in a VLE while teaching can be both stressful and time-consuming for trainers, textual information can be massive, and manual analysis can lead to biased interpretative issues (Yadegaridehkordi et al., 2019). In recent years, with the spreading use of big data analysis, institutions have been able to apply increasingly automatic computational techniques to analyze participants’ data. One of the techniques that has emerged is sentiment analysis (SA) or opinion mining, which uses artificial intelligence to analyze textual data in natural language with the aim of interpreting the emotions expressed by a group of participants (Barrón et al., 2020). SA of text input has been characterized as a nonintrusive and behavioral manner of emotion measurement (Feidakis, 2016).

In education, different SA methods have been developed as a proxy measure in real time of the emotional climate of a group, as well as to assess the quality of the trainer’s feedback (Yadegaridehkordi et al., 2019). SA has also been used to improve the understanding of educational processes, study participants’ satisfaction (Kastrati et al., 2021; Mite-Baidal et al., 2018), and make performance and dropout predictions (Iglesias-Estradé, 2019). However, these methods use a collection of textual data and have paid little attention to possible differences of communication because of personal characteristics of participants, such as cultural differences, language barriers, age and gender (Yadegaridehkordi et al., 2019).

It is necessary not only to develop methods that help trainers to process the huge amount of textual data from the VLEs, but also to develop inclusive, sensible, and reliable tools which consider the possible differences in trainees’ communication while estimating the emotional climate of a group. In this research, we put the focus on the design and assessment of a SA gender-sensitive method as a proxy to characterize the emotional climate of two cohorts of teacher trainees in an online Master’s program in Spain.

## Background

### Formative assessment and emotional climate in teacher training

Formative assessment involves a process of verification, assessment, and decision-making with the purpose of optimizing the teaching–learning process (Pinger et al., 2018). Compared with final or summative assessment, it can increase both motivation and involvement of trainees and provide opportunities for the correction of errors (Silvers & Sarvis, 2020). Hence, formative assessment represents a learning experience itself, developing trainees’ responsibility, autonomy, and communication, thereby improving their capacity for self-reflection and academic achievement (Martínez et al., 2016). These formative assessment practices will be mimicked and implemented by teachers, following their experience as trainees (Hamodi et al., 2017). Moreover, the effective integration of formative assessment has additional potential because it can offer an appropriate structure for sustained meaningful interactions through the development of effective online learning communities (Sorensen & Takle, 2005), which can be of special interest for preservice and inservice teachers. 

Certainly, formative assessment can be easily adapted to the asynchronous nature of interactivity in online learning environments. Among others, discussion forums are common formative assessment format either in synchronous or asynchronous manner (Xiong & Suen, 2018) and can be useful for promoting trainer feedback, peer assessment and automated feedback.

The use of discussion forums as a tool for providing formative assessment is well-considered by participants in online learning environments (Gaylard Baleni, 2015; Ogange et al., 2018). However, despite participants’ positive attitudes towards the use of discussion forums, research evidences suggests that its use involves a significant workload for trainers in monitoring the online environment and subsequent discussions between students (McCarthy, 2017). This difficulty in digesting a large amount of textual data in a relatively short time can seriously jeopardize the quality of periodic formative assessment provided, despite the potential benefits of using discussion forums and similar formats to provide formative assessment.

Different studies have focused on how the affective domain can influence the learning process and therefore needs to be considered within formative assessment. Participants can share interesting insights into the course topics and provide their impressions and affective states during the course through online forums and debates (Moreno-Marcos et al., 2019).

The educational environment is a consistent determinant of students’ cognitive and affective outcomes (Fraser et al., 2021). In particular, the emotional environment, or the quality of social and emotional interactions in the classroom—between and among students and teachers—influences students’ achievement (Reyes et al., 2012). When an emotional climate is characterized by warm, respectful, and emotionally-supportive relationships (a positive climate), students perform better, partly because they are more emotionally engaged in the learning process and they enjoy it more (Reyes et al.). In Kashy-Rosenbaum and colleagues (2018) experimental study, academic achievement was significantly higher within classrooms characterized by positive emotional environment, but significantly lower within classrooms characterized with negative emotional environment.

Several limitations are also met when measuring emotional climate in online learning environments. Traditional instruments for measuring a group’s emotional climate are based on thoroughly-designed questionnaires aimed at retrieving a subjective report from each participant about his/her psychological state. These include the questionnaires developed by Fraser et al. (2021) for STEM classrooms or by Alonso-Tapia and Nieto (2019) for high-school students. Other traditional instruments use observational tools to capture the behavioral activity to infer the emotional climate, such as the Classroom Assessment Scoring System of Pianta and colleagues (2008). More recently, with the generalization of the use of sensors, implicit detection of emotion has involved measuring physiological data, such as heartbeat, facial expressions, etc. (Feidakis, 2016). However, none of these three approaches fits well in online learning environments. Firstly, the behavioral and measurement of physiological data is impractical. Secondly, because the asynchronous nature of online learning environments—even though some activities can be carried out synchronously—makes it futile to try to measure a group’s emotional climate at a particular moment in time.

Therefore, to leverage the benefits of formative assessment in trainees’ outcomes, it is necessary to develop automated tools to help trainers to process data, so that they can provide authentic real-time feedback based on the emotional climate of their group of trainees. At this point, textual data mining becomes a necessary tool for facilitating formative assessment in online learning environments.

### Using sentiment analysis (SA) to improve formative assessment in online learning environments

Sentiment analysis (SA) is defined as the computational field concerned with contextually mining of unstructured text documents (such as opinions, sentiments, attitudes, evaluations, or emotions) so that structured and insightful knowledge can be obtained and employed for different tasks (Mite-Baidal et al., 2018). In education, a recent literature review conducted by Kastrati and colleagues (2021) identified 92 studies on SA of students’ feedback in online learning environments which, in general, measured the comments of trainees concerning various aspects of the trainers’ role. Results highlight the need for standardized solutions and a focus on emotional expression and detection, because the field is rapidly growing.

Research on this topic has identified the existence of gendered patterns of communication in participants’ uses of VLEs, which is different for specific areas. For example, in Sun and colleagues’ (2020) research on online technology communities, the frequency of female users expressing positive emotions was higher, and also male participants were more likely to express impatience and dissatisfaction in the process of technical learning. Similar patterns were found in the analysis of female and male posts in social media (Çoban et al., 2021). These authors also found that male users posted more positive messages as they grew older, while the reverse was observed for females. Therefore, age would also influence the gendered patterns observed. In other areas, such as health communities, female users are more likely to seek emotional support in health communities (Liu et al., 2018) and express more-negative emotions than male users, especially the expression of anxiety and sadness.

Females usually interact more in the VLE than their male peers (Oreski & Kadoic, 2018; Van Horne et al., 2018) and they usually participate to a lesser extent in the proposed activities with contributions that integrate fewer mistakes (Kickmeier-Rust et al., 2014). Shapiro et al. (2017) observed that females expressed more-negative views about their progress and self-perceived evaluation. These findings are aligned with previous results in the literature showing that female students usually underestimate their abilities compared with their male peers with similar achievements, especially in the areas of mathematics, computing, and social sciences (Huang, 2013). However, no previous literature was found on the use of SA to explore gender differences in the communication patterns—and sentiment expression—of trainees in higher education, which could jeopardize the use of SA as a formative generalized assessment tool.

In summary, previous studies identify a need for developing more-accessible ways of performing SA as a tool for measuring the emotional climate of a group in online learning environments. Moreover, although other personal factors might influence how students express themselves (i.e., age, race, and socioeconomic level), there is a need to integrate a gender perspective when developing SA techniques not only to develop a deeper understanding of participants’ interactions, but also to design more-inclusive tools and implement a better and more-targeted formative assessment.

## Method

An explanatory case study with mixed methods (Simons, 2009) was implemented in developing a SA technique for characterizing the emotional climate of a group of trainees in an online learning environment and assessing its validity while considering participants’ gender, achievements, and satisfaction.

### Context of study

This study took place in a Master’s program in Educational Technology in Spain, where preservice and inservice teachers engage to develop their professional competencies related to the use of digital technologies in their professional practice. The two-month subject *Data Collection and Analysis Tools and Techniques* was chosen because it had a previous formative assessment design; trainee teachers had continuous feedback on their achievement at an individual level. Moreover, this subject has been traditionally perceived as the most difficult of the program. Consequently, we anticipated that the evolution of participants’ perceptions would be more evident in their messages on the VLE compared with other subjects.

### Aim and research questions

With the aim of explaining gender differences in relation to sentiment expression, learning achievement and satisfaction with a teacher training program as a first step for using SA to characterize the emotional climate, the following research questions were studied:

RQ1. What is the relationship between participants’ sentiment expression in the VLE, learning achievement, and gender?

RQ2. What is the relationship between participants’ sentiment expression in the VLE, satisfaction with the course, and gender?

### Instruments and data retrieval

**Fig. 1:**
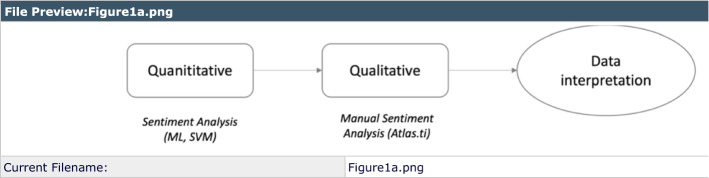
Sequential explanatory design adapted from Simons (2009), with quantitative sentiment analysis preceding qualitative study of sentiments

Four videoconferences were conducted during the course as part of the instruction (VC1-VC4) in a Moodle course (Fig. 1) together with four evaluation activities. The first activity (EA1) was an introductory discussion forum on educational research during which the trainer posed some questions and students interacted (see Appendix 1 for examples of these interactions). Three more forums were opened to give space for participants’ interaction and sharing of doubts and feelings about the three scored activities, which comprised a quantitative analysis exercise (EA2), participants’ understanding of qualitative analysis techniques (EA3), and an individual practical activity for which students had to develop and present a proposal of data retrieval and analysis for their Master’s thesis (FA). In these three forums, students voluntarily posted related questions, comments, and other information to facilitate the SA process. Participants’ scores in EA1 were weighted 10% for the final score, 25% each for EA2 and EA3, and 40% for FA.

**Fig. 2:**
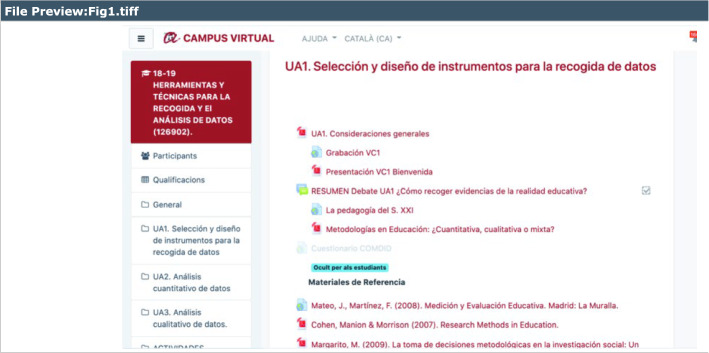
Screenshot from first unit in the VLE (Moodle) of the subject, UA1 Discussion forum

The VLE was used to centralize all trainer-trainee and trainee-trainee interactions. In order to make sure that trainees interacted only through the VLE (Moodle, see Fig. 2), at the beginning of the course, the trainer recorded and shared a video and a text welcome message that stated that the preferred communication channel for participant-teacher and participant-participant interactions would be the Moodle forums.

**Fig. 3:**
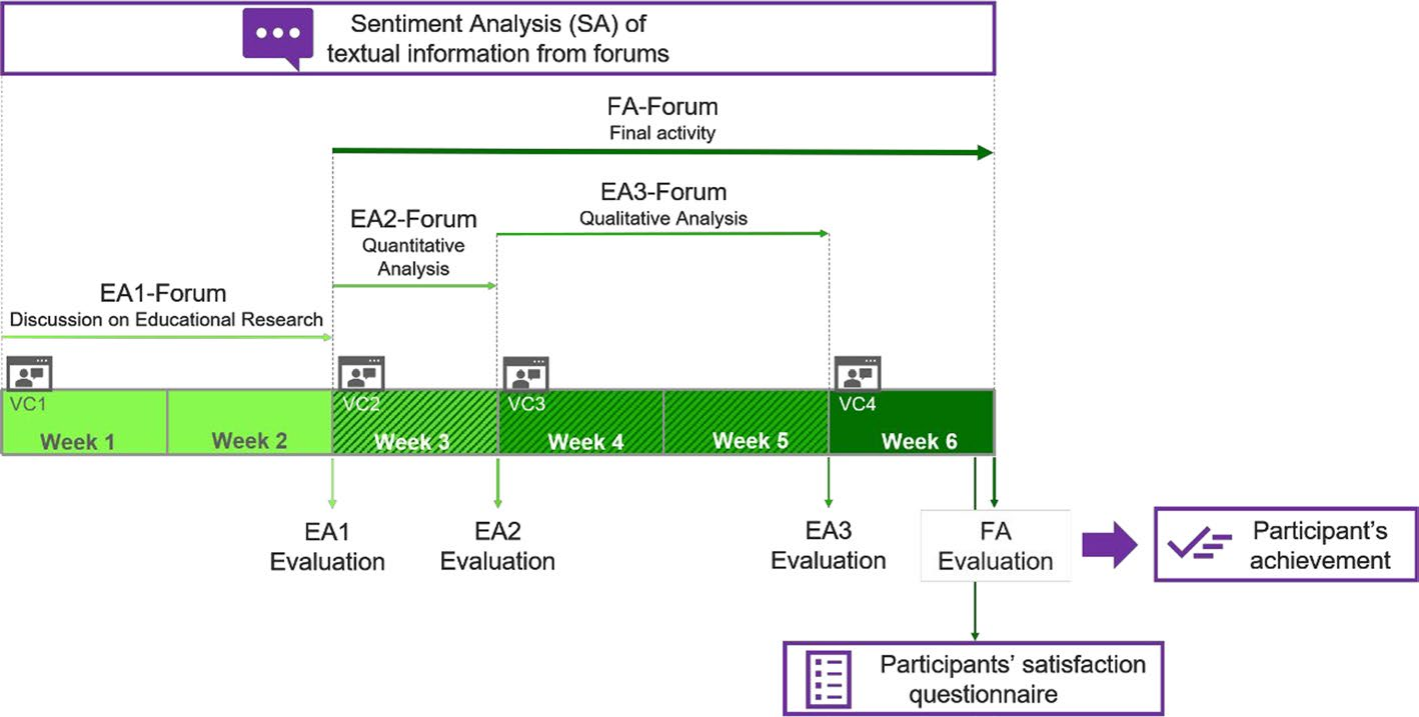
Time schedule of the learning activities and questionnaires (EA: evaluation activities; EA1: 10% of the final scoring; EA2: 25% EA3: 25%; Final Activity: FA: 40%; VC: videoconference)

Data retrieval was divided into the following three stages. First, textual data from VLE was studied using Sentiment Analysis. Participants’ messages on forums were directly downloaded from the VLE together with Moodle ID to anonymize the data while tracking the answers. Second, an online questionnaire was distributed to participants immediately before the end using two open-ended questions (Appendix 2). Participants were asked about their satisfaction with the program. This information was gathered to explain the relationship of participants’ previously expressed sentiments with their final satisfaction to qualitatively assess the validity of SA. Additional questions were added at the end of the questionnaire to ascertain participants’ gender, and other professional data. Third, participants’ learning achievement in the course was built from their grades in the four different evaluations described below (Fig. 2). Trainees’ grades were used to assess the predictive capacity of SA method (Fig. 3).

Regarding ethical aspects, all participants were informed about the aims of the research, subsequent treatment of the anonymous data gathered, and the voluntary nature of their participation. All analyses were carried out after the completion of the course to avoid misunderstandings with the final grading.

### Sample

The sample for this case study was made up of the cohorts of participants of the 2018–19 and 2019–20 courses. The trainer, the syllabus, and the learning tasks were the same for the two cohorts, but differences in participants’ interactions on forums and satisfaction were not considered I terms of the characteristics of the subject in each academic course. However, results were analyzed and presented separately to increase reliability of SA. Of the total of 61 trainees enrolled in this subject during the 2018–2019 and 2019–2020 years, 10 people (16.39%) dropped out of the subject (5 women and 5 men). The final sample comprised 48 students (26 women and 22 men; mean age 33.50 years; SD = 8.69 years) who answered the questionnaires and participated in all evaluation activities. Differences in the two cohorts are displayed in Table 1.

**Table 1:** Description of participants in the two cohorts

Cohort	Number of participants	Proportion of women (%)	Age Mean (SD)	Teaching experience (median)*
2018–19	31	42%	32.47 (8.89)	1
2019–20	17	59%	34.06 (8.46)	2

*Teaching experience was measured as an interval variable. 1: less than 5 years, 2: between 5 and 10 years

### Data analysis

The data analysis for the study involved both quantitative and qualitative measures. Support Vector Machine was used in SA; content analysis methods were used to interpret participants’ responses to the satisfaction questionnaire with the software Atlas.ti 8 (ATLAS.ti Scientific Software Development GmbH) for Windows to validate the effectiveness of the SA. Finally, a correlational analysis was conducted using JASP V0.11.1^1^software.

#### Descriptive sentiment analysis

Sentiment expressions from textual data were rated as positive, negative and neutral in order to train and run the machine learning process and further analysis (Pang & Lee, 2008). In particular, to retrieve the sentiment polarity from a student's post in the VLE, we used a support vector machine (SVM) because SVM is the machine learning strategy that gives the best performance in the SA field (Oloruntoba & Akinode, 2017). A code written in Python language within the Anaconda Navigator 1.9.12 environment has been developed, based on Garrido’s (2015) previous work. In order to train and test the SVM using a preliminary set-up to test the SVM’s capacity to distinguish between positive, neutral, and negative information with a set of previously-tagged messages and their progressive improvement), we took advantage of TASS corpus, a set of tweets previously classified in different sentiment polarity in Spanish (Martínez-Cámara et al., 2016; Villena-Román et al., 2013, 2014, 2015;). A linear kernel was selected to classify the documents based on the fact that sentiment analysis corpus was classified in two different polarities. To preprocess text data, CountVectorizer tokenizer was selected. Furthermore, a pipeline was applied to get the best parameters for prediction. Finally, the parameters selected were as follows: { 'vect__max_df': 0.5, 'vect__min_df': 50, 'vect__max_features':1000, 'vect__ngram_range': (1, 1), # unigramas 'cls__C': 0.2, 'cls__loss': 'squared_hinge', 'cls__max_iter': 1000}.

#### Complementary qualitative analysis of participants’ satisfaction with the program

To examine the interpretative power of the SA technique developed, a complementary qualitative analysis was undertaken to assess the relationship of the evolution of the sentiments expressed by participants with their satisfaction with the program. Content analysis methods were used to interpret participants’ satisfaction with the program retrieved from the final online questionnaire, following McMillan and Schumacher (2010). The aim of this phase was to transform opinions into a condensed form of information (categories) that could facilitate the subsequent comparison with results of SA and contrasts by gender. The process of defining and refining the opinion categories was based on a mainly-inductive cyclical process to optimize the total number of categories considered and the meaning given to each one. Two researchers participated in the refinement process to assess the consistency of the analysis.

Opinion categories were defined with a positive or negative direction for contrasting with participants’ results in the SA and were structured into two families: *Master’s training capacity for professional development* (10 categories); and *Master’s managing capacity* (3 categories). Additionally, identification categories (i.e., gender and teaching experience) were applied to participants’ satisfaction. After matching opinion categories to identification categories, the corresponding χ^2^ statistic was calculated for two nominal variables to assess possible differences of participants’ satisfaction according to their gender. From the analysis of the total frequencies and gender differences, relevant opinion categories were identified and considered for the purposes of this study. Therefore, relevant opinion categories were defined as the ones with a minimum frequency of 10 participants or with a significant difference in the gender distribution. The final categories and their frequencies are displayed in the results section.

#### Validating SA with correlational analysis

SA from trainees’ messages on forums and debates was correlated with achievement in all the learning tasks (AE1, AE2, AE3 and FA) and with the qualitative analysis of participants’ satisfaction in order to validate the effectiveness of the SA process. First, a parametric correlation analysis identified the relationship between sentiment expression and task performance (continuous data). According to prior studies, this relation would be positive and significant, showing that the more positive the sentiments expressed in the VLE, the better the learning achievement (Iglesias-Estradé, 2019). Second, a nonparametric correlational analysis (Pearson correlation) was conducted to identify the direction and strength (statistical significance) of the relationship of sentiment expression and the nominal variable of gender (Cohen et al., 2013). The contrasts were carried out with a significance level of 95% and with JASP V0.11.1^2^software.

## Findings

A first description of the total number of posts and participants analyzed for the SA is displayed in Table 2 to present the results for RQ1 (relationship between participants’ sentiment expression in the VLE, learning achievement, and gender).

**Table 2:** Number of posts analyzed and total participants in each forum

Year	Task	Total posts	Total participants
2018–2019	FA Forum	361	21
	AE1 Debate	2561	196
	AE2 Forum	176	44
	AE3 Forum	83	24
2019–2020	FA Forum	92	8
	AE1 Debate	1752	125
	AE2 Forum	293	35
	AE3 Forum	133	28

The classification system used in this analysis was a ROC curve, which is a graph that shows the performance of a classification model at all classification thresholds. This curve represents two parameters:

Real positive tax: TPR = TP / (TP + FN).

False positive tax: FPR = FP / (FP + TN).TP: true positives; FN: false negative; FP: false positive; TN: true negatives.

To calculate the points of the ROC curve there is an efficient algorithm based on classification that provides this information, namely, area under the ROC curve (AUC). AUC measures the entire two-dimensional area below the entire ROC curve from (0.0) to (1.1)*.* The best AUC parameter from which the analysis was made was 0.939, evidencing a correct performance of the classifier to distinguish between all positive posts (e.g. *Leídos parte de los documentos y contenido de la EA1 veo que he acertado, y me alegro enormemente…* in EA1, translated as *Having read part of the documents and content of the EA1, I see that I have been right, and I am extremely happy…*), neutral posts (e.g. *Exactamente esa es la duda que sigo teniendo después de mirar el PDF* in EA2, translated as *That is exactly the question that I still have after looking at the PDF*) and the negative posts (e.g. *Mi compañero y yo estamos un poco atascados, pensábamos que lo habíamos entendido, pero no estamos seguros* In EA2, translated as *My partner and I are a bit stuck, we thought we understood, but we are not sure*). The closer the AUC value is to 1, the higher is the reliability of the classifier in distinguishing between the positive and negative posts.

### Relationship between SA and participants’ achievement (RQ1)

Results of participants’ achievement in the different learning tasks are shown in Table 3, together with the sentiment expression of their contributions in the correspondent discussion forums. SA was measured for each forum separately; all messages in each forum were analyzed and the rate of positive messages was extracted according to the SVM results after it was trained with Spanish TASS corpus. Although sentiment expression in the forums showed a positive correlation with achievement in the course, it was not statistically significant at a confidence level of 95% based on results of the Pearson correlation coefficient (*r* = 0.23; *p* = 0.75).

**Table 3:** Task achievement and sentiment expression for each learning task (Task achievement is reported as the average percentage score of the group in each task. Sentiment Expression reflects the positivity of the messages in each forum, ranging from 0 (total negative) to 1 (total positive))

Year	Task	Sentiment Expression (/1)	Task Achievement (%)
2018–2019	AE1	0.7275	79.69
	AE2	0.7216	72.50
	AE3	0.7831	71.94
	FA	0.8670	77.07
2019–2020	AE1	0.7826	67.23
	AE2	0.7089	70.34
	AE3	0.7143	69.32
	FA	0.7534	66.29

### Relationship between SA and participants’ gender (RQ1 and RQ2)

Table 4 displays the percentage of females who participated in each forum and the sentiment expression retrieved from the messages. To provide consistency with the second research question, the percentage of women’s participation in every forum was used instead of general gender percent. Results show that SA and women’s participation was negatively correlated (*r* = −0.73; *p* < 0.05), with women participating less than men and expressing more negative sentiments.

**Table 4:** Sentiment expression and gender participation in each forum (Women's participation rate is the percentage of women who participated in each forum)

Year	Task	Sentiment Expression (/1)	Women participation rate* (%)
2018–2019	AE1	0.7275	21.43
	AE2	0.7216	11.36
	AE3	0.7831	8.34
	FA	0.8670	9.52
2019–2020	AE1	0.7826	37.50
	AE2	0.7089	57.14
	AE3	0.7143	46.43
	FA	0.7534	36.00

To answer RQ2, results of participants’ satisfaction are summarized in Table 5 together with the absolute frequency and the percentage of women in the category,. A complete version of the table can be found in Appendix 3 with excerpts. Only relevant categories with a minimum frequency of 10 participants or with a significant difference in the gender distribution were considered in our study. Participants reviewed negatively the program’s training capacity (e.g. *Teacher’s feedback (…) is not provided in a very fast way. Chats are answered quite quickly, but ratings take a longer time*), summarized in the practicality and assessment categories. Satisfaction with the program’s managing capacity was mostly positive (e.g. (I appreciate the Master's) *for its value in learning more about education and getting more points to get a stable job*), summarized in the certification, adaptation, and coordination and planification categories.

**Table 5:** Categories used in the analysis of participants’ satisfaction with the Master’s, frequency of categories, and the percentage of women who provided these answers (DT: Digital Technologies)

Category	Description	Frequency	Percentage of women (%)
Practicality	Practical applicability of the content of the Master's is negatively valued	15	73
Coordination and planning	The coordination of the Master's and the planning of the subjects is negatively valued (e.g. overlapping content, not considering participants’ previous knowledge, periods of excessive workload, little time for deliveries…)	25	56
Assessment	Assessment (either summative or formative) is valued negatively because of the quality of feedback, the slowness of the response, or the grading system	14	36
Certification	The Master's is positively valued as a tool for certifying the training received and improving one’s CV for competitive examinations	6	100
Adaptation	The Master’s is positively valued for its capacity to adapt to personal situations	21	38

The proportion of women who positively rated the potential of the program’s certifying (100%) was found to be statistically different from the sample, χ^2^ (1, 48) = 5.333, *p* = 0.021. The proportion of women who negatively rated the assessment (36%) was slightly statistically significant, χ^2^ (1, 48) = 3.387, *p* = 0.066. On the other hand, the proportion of women who value the adaptability of the program was significantly lower than the proportion of male colleagues, χ^2^ (1, 48) = 5.000, *p* = 0.025.

## Discussion

The purpose of this study was to explain the application of a SA technique to identify gender differences in relation to sentiment expression as a proxy measure of the emotional climate of a group in an online learning environment. The measures of sentiment expression were contrasted with participants’ learning achievement and satisfaction with the program as a first step for validating this technique. From the analysis, three main themes emerged, which are discussed below.

### Positive emotional climate is related to participants’ gender and the design of activity (RQ1)

Sentiment expression, in general, was rated as positive, which is similar to Buckingham Shum and Ferguson’s (2012) results. However, our results revealed that women posted significantly-more negative messages than their counterparts, which supports previous findings by Shapiro et al. (2017) that female participants expressed more-negative views about their progress and self-perceived evaluation in online environments, but contradicts findings of Çoban et al. (2021) and Sun et al. (2020). Hence, the effect of participants’ gender on emotional climate could be different according to the area, which reinforces the need for integrating a gender perspective in SA.

When analyzed in detail, ratings within the diverse forums were different, suggesting different implications for participants in different forums and for the emotional climate of the group. Ratings in the discussion forum (EA1) were a highly positivite compared with other forums (around 73% to 78% for the 2018–19 and 2019–20 cohorts, respectively). According to the results and prior literature, this positive emotional climate can be related to the significant emphasis on the collaborative nature of the activity, as well as by the fact that both groups were composed mostly by females. Hence, females tend to enjoy more-collaborative activities compared with their male peers according to Atwood-Blaine and Huffman (2017). These findings also explain why a more-positive climate was found for the 2019–20 cohort for which women were in the majority. Therefore, and adding to previous research results, participants’ gender distribution could be a relevant factor to consider when designing collaborative learning environment in online courses.

Results of the positive climate in the first forum were also related to how the associated task in the forum was conducted and its perceived initial level of difficulty. Certainly, collaboration in an online environment can be a socially- and emotionally-demanding task which not always results in a positive emotional climate, as Bakhtiar et al. (2018) describe. In our case, this forum was designed with an easy entry-level to the course and participants mobilized their own personal resources and previous knowledge without difficulty to complete the designed task. Therefore, the overall preparation in terms of prior knowledge of topics and self-regulation were appropriate, which is key factor identified by Bakhtiar and colleagues for generating a positive socio-emotional climate in online collaborative learning environments. These results support the relevance of implementing introductory collaborative activities with an easy entry level in order to build a community with a positive emotional climate.

In the other forums (EA2, EA3, FA), participants only shared doubts and questions around the individual learning tasks, generating a different and less-supportive group dynamic. In particular, sentiment expression in the forum related to the quantitative analysis activity (EA2) was linked with the most-negative climate for both cohorts. This task is usually considered as the most difficult by trainee teachers, with participants perceiving themselves as less competent and, consequently, sharing less-positive sentiments, in line with previous results (Djudin, 2019). Hence, participants’ preparation or self-regulation prior to the task was perceived as insufficient by trainees compared with the assignment, thus hampering a positive collaborative experience, in line with Bakhtiar et al. (2018). These results show how the design of well-adjusted teaching–learning sequences with appropriate scaffolding also contributes to a positive climate in online learning environments, where participants feel that they are prepared to tackle the next step in the learning pathway.

The relevance of participants’ self-regulation skills in creating positive climates in online learning environments is also evidenced by the results of SA in the two cohorts. Participants in the 2018–19 cohort became more positive as they progressed in the subject, unlike the 2019–20 cohort participants who scored lower in the SA of the final activity (FA) compared with EA1. This trend could be related with participants’ teaching experience and age, because the 2018–19 cohort was more novice and younger compared with the 2019–20 cohort. Çoban et al. (2021) highlight that older female participants–a majority in the studied course–express more-negative messages. Following this interpretation, the more-experienced cohort would have initially higher self-confidence, which would have not been satisfied with the development of the subject’s results—explaining the decrease in the positiveness of the climate. Conversely, the more-novice cohort would have less self-confidence initially but grow it during the subject–explaining the increase in the positiveness for this group. In summary, age would also act as a relevant factor influencing the climate of the group, mediated by possible differences in participants’ self-regulation skills in line with Bakhtiar et al. (2018).

### Sentiment expression is related to participants’ achievement, but not significantly (RQ1)

Previous literature shows how a positive climate in the learning environment is related to better student achievement (Fraser et al., 2021; Iglesias-Estradé, 2019; Reyes et al., 2012). Our results support these findings, because the sentiment expressions of participants were correlated with learning achievement, although not a significant level. Therefore, sentiment expression as a proxy of emotional climate cannot be used to exactly predict participants’ achievement, but it could guide trainers to foresee how participants broadly act in a learning task and, therefore, to use these SA results for tuning and improving the quality of the guidance during the course.

### Gender differences are found in participants’ satisfaction with the program, evidencing a connection with their sentiment expression (RQ2)

There were gender differences in participants’ satisfaction with the program: men were more critical with the assessment and follow-up approach and the adaptation of the program to their own personal circumstances. These differences seem to support gendered patterns found in SA results related to the emotional climate, with female participants posting more negative messages than their counterparts. Male participants are considered to hold more-positive self-perceptions compared with women (Shapiro et al., 2017) but, because no gender differences were found in participants’ achievement, we interpreted male participants’ more-critical opinions in the final satisfaction questionnaire as suggesting disappointment with the program caused by a mismatch with their self-perceived capacities (Table 3). Conversely, female participants, who had expressed fewer positive sentiments in all forums but had achieved similar results than their male counterparts, would express a higher level of satisfaction with their achievements. The coherence between these results constitutes a first step in the validation of the effectiveness of the SA developed as a proxy of emotional climate.

## Conclusions and implications

This case study has presented a technique for performing SA of participants’ interactions in discussion forums extracted from a VLE, developed for Spanish-speaking participants in an online teacher training course. Results highlight the power of SA as a proxy measure of emotional climate, acting as an indicator of the perceived difficulty of the demanded task in collaborative activities and, subsequently, of participants’ preparedness and developed self-regulation skills for addressing it. Emotional climate can signal potential low task achievement of the group and can be useful for teacher trainers when helping the group before the submission of each activity, especially in better tuning the demands in a teaching–learning sequences.

The observed gender differences reinforce the need for integrating a gender perspective in the development of SA methods and interpreting the emotional climate of a group. In our research, female participants expressed more-negative sentiments than their counterparts and felt more confident in collaborative learning spaces. Male participants showed more-positive self-perceptions but would possibly experience disappointment with the program at the end. The proposed SA method has been found to be sensitive to these gender differences. These findings lay the ground to continue exploring the expansion of the potential of SA results as a comprehensive and real-time measurement of trainees’ emotional climate and leveraging the potential of online learning environments for tailoring trainees’ needs.

Because the VLE Moodle has widespread use in higher education, this proposal could be easily replicated in other subjects, courses and/or institutions, making automatization of SA in higher education feasible. However, procedures for extracting textual contents are specific to the format of each VLE, as well as to how the system administrator organizes the tasks. Hence, automating SA would be favored between courses using the same VLE, which is usually the case within the same institutions. Otherwise, an adaptation of the natural-language processing code for retrieving the forum text would be needed for different VLEs (Villena-Román et al., 2015).

Similarly, the creation of a tool to inform trainers about groups’ sentiment expression would be again restricted to the characteristics of the VLE, which could be tailored or developed specifically. However, we argue that these two aspects represent minor issues for large institutions, compared with the interpretative potential and the understandable results retrieved by this technique. Finally, because of the novelty of our proposal with Spanish-speaking participants, a greater number of analyzed messages in Spanish would help to refine the analysis and contribute with more insightful information for better understanding participants’ learning needs and designing more gender-inclusive spaces. The interpretative potential, its degree of automatization, and the type of results retrieved, complemented with the qualitative analysis, make this assessment method easy to use by trainers, who could benefit from real-time group feedback on students' emotional climate.

## Message on EA1: Discussion forum activity:

“Buenas tardes X.

Ese mismo artículo pone de manifiesto que los diseños de investigación basados en metodología mixta vienen adquiriendo relevancia en el campo de las Ciencias Sociales como base para entender y modificar diferentes aspectos asociados al campo educativo (Pereira, 2011). Esto es debido a que el uso simultáneo de ambos métodos (cuantitativo y cualitativo) mejora la capacidad de comprensión de los fenómenos que se están estudiando, máxime cuando tenemos que interpretar campos tan complejos como la diversidad del ser humano.

Un saludo.”

[Good afternoon X.

That same article shows that research designs based on mixed methodology have been gaining relevance in the field of Social Sciences as a basis for understanding and modifying different aspects associated with the educational field (Pereira, 2011). This is because the simultaneous use of both methods (quantitative and qualitative) improves the ability to understand the phenomena that are being studied, especially when we have to interpret fields as complex as the diversity of the human being.

A greeting.]

## Message on EA2: Doubts forum on the quantitative analysis forum

“Buenos días, yo también estoy como mis compañeros, con dudas y un poco perdida con esta actividad. He estado investigando estos días para intentar hacerlo pero no consigo avanzar.

X, podrías hacer una videoconferencia para resolver dudas? La actividad hay que entregarla el martes y veo que no llego.

Un saludo”.

[Good morning, I am also like my colleagues, with doubts and a little lost with this activity. I have been investigating these days to try to do it but I cannot advance.

X, could you make a videoconference to answer questions? The activity must be delivered on Tuesday and I see that it did not arrive.

a greeting].

## Appendix 2

Questions used in the online questionnaire to gather data about participants’ final satisfaction:

- What do you think are the main potentialities of this master's degree? (*participants’ satisfaction)*
- What do you think are the main weaknesses of this master's degree? (*participants’ satisfaction)*

## Appendix 3

Complete description of the categories used in the analysis of participants’ satisfaction with the Master’s (DT referrs to Digital Technologies.

**Table:**

	Category (Absolute frequency)	Description	Percentage of women
*Master’s training capacity for professional development*	- Practicality (15)	Practical applicability of the content of the Master's is negatively valued Quotation example: *Too theoretical*	73%
	- Coordination and planning (25)	The coordination of the Master's and the planning of the subjects is negatively valued (e.g. overlapping of contents, not taking into account participants’' previous knowledge, periods of excessive workload, little time for deliveries…) Quotation example: *The deadlines are inadequate, considering that some of us have work and do other things that prevent us from dedicating 5 h a day to the Master’s tasks and projects*	56%
	- Assessment (14)	Assessment (either summative evaluation or formative assessment) is valued negatively due to the quality of the feedback, the slowness of the response, or the grading system Quotation example*: Teacher’s feedback (…) is not provided in a very fast way. Chats are answered quite quickly, but ratings take a longer time*	36%
	+ Certification (6)	The Master's is positively valued as a tool for certifying the training received and improving one’s CV for competitive examinations Quotation example*: (I value the master for) its value to learn more about education and get more points to get a stable job*	100%
*Master’s managing capacity*	+ Adaptation (21)	The master’s is positively valued for its capacity to adapt to personal situations Quotation example*: The course is exclusively online, which makes it easier to carry it out*	38%

## Footnotes

1 JASP Team (2020). JASP (Version 0.13.1) [Computer software].

2 JASP Team (2020). JASP (Version 0.13.1) [Computer software].
